# Pure Red Cell Aplasia Following Interleukin-2 Therapy

**DOI:** 10.1177/2324709616643991

**Published:** 2016-04-11

**Authors:** Janice P. Dutcher, Wen Fan, Peter H. Wiernik

**Affiliations:** 1 Cancer Research Foundation of New York, Chappaqua, NY, USA; 2 Mt Sinai-West Hospital, New York, NY, USA

**Keywords:** red cells, aplasia, interleukin-2, immune mediated

## Abstract

A 61-year-old woman with metastatic renal cell carcinoma underwent systemic treatment with high-dose interleukin-2 (IL-2). Anemia requiring transfusion of 1 unit of packed red blood cells (PRBCs) was required during the second week of IL-2 therapy. One month following completion of high-dose IL-2 treatment, she was hospitalized for severe, symptomatic anemia and received 5 units of PRBCs. She was referred back for evaluation. A complete hematologic evaluation was performed including antiviral serology, evaluation for hemolysis, complete iron studies, and finally bone marrow aspiration and biopsy. The diagnosis was pure red cell aplasia, and no inciting viral cause could be ascertained. She required PRBCs for 5 months following IL-2 therapy. It was concluded that IL-2 was the cause of her red cell aplasia. This subsequently resolved spontaneously, and she had normal hemoglobin and hematocrit, respectively, 1 and 2 years after treatment.

## Introduction

The diagnosis of pure red cell aplasia (PRCA) can only be made when the following criteria are met: (*a*) normocytic, normochromic anemia; (*b*) low to zero reticulocytes; (*c*) normal white blood cell count and platelet count; and (*d*) a bone marrow that is normocellular with little to no erythroid precursors but normal myelopoiesis, lymphopoiesis, and megakaryocytopoiesis.^1^

Although PRCA is a rare phenomenon, it has been well-characterized in relation to certain specific inciting causes. These include the most rarely reported, resulting from an acute viral infection such as hepatitis C,^2,3^ or more frequently reported, exposure to parvovirus B19, in which the parvovirus directly attacks the pro-erythroblasts.^4-6^ Other frequent occurrences are related to autoimmune disorders, which produce erythroid destruction related to both antibodies (lymphoproliferative disorders, systemic lupus erythematosus, rheumatoid arthritis)^7,8^ and to T-cell dysfunction (thymoma).^9,10^ Within the past 15 years, a new phenomenon of PRCA has been observed in patients who received specific types of exogenous erythropoietin as treatment for chronic anemia and who developed antibodies directed toward erythropoietin.^11-13^

Attempts to modulate severe PRCA have primarily focused on modulating the immune-mediated process through immunosuppressive therapy, such as corticosteroids, rituximab, cyclosporine, or azathioprine. However, once developed, unless the inciting cause can be reversed or controlled, there may remain chronic anemia, particularly in the form induced during erythropoietin therapy.^11-13^

We report here a case of reversible PRCA that developed as a result of therapy with high-dose interleukin-2 (IL-2) for metastatic renal cell cancer (RCC). To our knowledge, this is the first report of PRCA as a result of IL-2 therapy, despite clear documentation of other autoimmune phenomena occurring subsequent to IL-2 therapy, including hypothyroidism, reactivation of inflammatory bowel disease, psoriasis,^14-19^ and activation of occult rheumatoid arthritis (J. P. Dutcher, personal communication).

## Case Report

A 61-year-old woman presented with shortness of breath and leg edema but was found to have no evidence of pulmonary emboli on computed tomography (CT) of the chest, but had a 1.4-cm left lower lobe pulmonary nodule. There were also sub-centimeter enhancing foci in the liver, of indeterminate significance. The shortness of breath subsided and was felt to be an allergic reaction to medication. Five months later, she underwent a follow-up CT scan, which demonstrated bilateral pulmonary nodules, the largest being 1.8 cm in the left lower lobe, slightly increased from the prior scan, and the liver lesions also appeared somewhat larger. One month later, she underwent a CT-guided biopsy of the dominant left lung nodule, and pathologic evaluation revealed clear cell RCC. She had a history of a nephrectomy 10 years prior, for a 3-cm clear cell RCC, with all margins negative. She had had annual follow-up imaging until the past 5 years when insurance issues interfered with her follow-up.

She was referred for high-dose IL-2 therapy, which was initiated 4 months after the diagnostic lung biopsy. Prior to IL-2 therapy, she underwent prescreening, which included negative serology for hepatitis A, B, and C and for human immunodeficiency virus (HIV). She had no significant other medical problems and described no recent or ongoing infections. She had no history of prior transfusions. She was not symptomatic from her metastatic RCC, and her performance status was Karnofsky 100%. She was therefore deemed an excellent candidate for high-dose IL-2 therapy. She received the standard regimen of IL-2 at 600 000 U/kg/dose, every 8 hours, for up to 14 doses, weeks 1 and 3. She received 10 doses during the first week and 6 doses during the second week. Her course was complicated by the expected toxicities of hypotension requiring vasopressors, systemic inflammatory response syndrome, mild thrombocytopenia, and elevation of liver function tests. All toxicities resolved after stopping IL-2. She had an episode of atrial fibrillation for a few hours on the last day of the last week, which resolved spontaneously. She received standard premedications with oral antinausea, antacid, and antibiotic medications during the administration of IL-2 (Table 1). None of these medications have been associated with PRCA.

**Table 1.** Medications.

Medication During Interleukin-2 Therapy	Known Hematologic Toxicity
Acetaminophen	Rare
Indomethacin	Platelet dysfunction
Cefalexin	Thrombocytopenia
Neosynephrine	Agranulocytosis
Morphine	None known
Famotidine	Pancytopenia, granulocytopenia
Diphenoxylate/atropine	None known
Lorazepam	None known
Gabapentin	Thrombocytopenia

Prior to starting IL-2, her hemoglobin (Hgb) was 11.6 g/dL with a hematocrit (Hct) of 35.7%. Prior to starting the second week of IL-2, her Hgb was 8.9 g/dL and Hct was 26.8%, with mean corpuscular volume (MCV) of 89.3, consistent with anemia of chronic disease. White blood cell and platelet counts were consistent with IL-2 treatment, 13.8 × 10^3^ and 473 × 10^3^, respectively. Unusual for this amount of administered IL-2, she did require a transfusion of 1 unit of packed red blood cells (PRBCs) during the second week of IL-2, for an unexpected continued fall in Hgb to a value of 7.0 g/dL with a normal MCV. On further investigation, there was no evidence of bleeding. At home, following discharge from the second week of IL-2 therapy, she had mild toxic encephalopathy manifested as agitation, which responded to lorazepam for 3 days, and she had prolonged pruritus that was treated with gabapentin, begun after her discharge from the second week of IL-2. Additionally, at home following IL-2 therapy, she noted the development of a number of skin lesions, some raised, similar to skin tags, one as large as 3 cm, but they mostly resolved by 6 weeks following IL-2 administration.

One month after discharge, the referring physician reported that the patient was hospitalized because of weakness, shortness of breath, and dizziness, and was found to be severely anemic with no evidence of hemolysis or bleeding. At that time her Hgb was 4.6 g/dL, and her Hct was 12.9%, with a normal MCV. Her serum bilirubin was 0.3 mg/dL, lactate dehydrogenase was 147 U/L, serum iron 200, total iron binding capacity 328 with iron saturation of 61%, and ferritin of 780 ng/mL. Her white blood cell and platelet counts were within normal limits. She received transfusions of 5 units of PRBCs. She did not recall any prior episodes of anemia during pregnancy or prior to/during recovery from her nephrectomy 10 years previously, and had never been transfused prior to the transfusion during the second week of high-dose IL-2.

Further evaluation again revealed negative serology for hepatitis A, B, and C and negative serology for cytomegalovirus. Serum folate was 14.94 ng/mL, and B_12_ was 474 pg/mL. Transferrin was 252 g/L. Coombs test was negative. Reticulocytes were 0.32%, haptoglobin was 345 (reference = 30-200). IgG and IgM antibodies to parvovirus B19 were 37 units (0.0-0.89) and 0.2 units (0.0-0.89), respectively, consistent with prior exposure, but reflecting no acute exposure. Erythropoietin level was <1.0 (reference = 3.7-31.5; Table 2).

**Table 2.** Laboratory Values.

Laboratory Parameter	Pre-IL-2	Post-IL-2			
		IL-2 Week 2	2 Months	4 Months	1 Year
WBC (× 10^3^)	5.3	13.8	6.1	6.4	7.5
Platelets (× 10^3^)	328	473	325	318	302
Hemoglobin (g/dL)	11.6	8.9	4.6	9.6 (untransfused)	13.3
Hematocrit (%)	36	27	12.9	29	40.3
MCV (reference = 80-100) (fmL)	91.8	89.3	84.2/86.3		95.7
Iron (µg/dL)			200	244	83
Ferritin (reference = 12-300) (ng/mL)			780 (transfused)	1601 (transfused)	600
Bilirubin (mg/dL)			0.3		
LDH (U/L)			147		
Epo (reference = 3.7-31.5)			<1		
Reticulocytes (%)			0.32		
Hepatitis A Ab		Negative			
Hepatitis B core IgM		Negative			
Hepatitis B Ag		Negative			
Hepatitis C Ab		Negative			
CMV Ab		Negative			
Parvovirus B19 IgM (0.0-0.89)			0.2		
IgG (0.0-0.89)			3.7		
Folate (ng/mL)			14.94		
B_12_ (pg/mL)			474		
TIBC (µg/dL)			328	240	
Iron saturation (%)			61		
Transferrin (g/L)			252		
Haptoglobin (reference = 30-200)			345		
Coombs			Negative		

Abbreviations: IL-2, interleukin-2; WBC, white blood cell; MCV, mean corpuscular volume; LDH, lactate dehydrogenase; CMV, cytomegalovirus; TIBC, total iron binding capacity.

**Figure 1.**
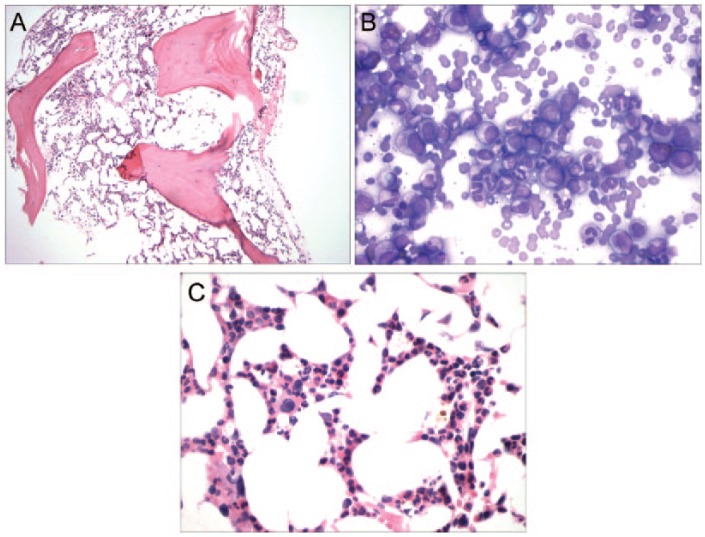
(A) Bone marrow biopsy (10× magnification): normocellular marrow for a 61-year-old person. (B) Bone marrow aspirate (60× magnification): normal myeloid maturation. (C) Bone marrow biopsy (60× magnification): pure red cell aplasia.

Because of persistent transfusion dependence, a bone marrow aspiration and biopsy was performed. This revealed a marrow with cellularity consistent with a 61-year-old woman, normal myeloid and platelet maturation, and complete absence of erythoid elements (Figure 1A-C). There was no morphological suggestion of myelodysplasia. It was felt that this may be PRCA induced by high-dose IL-2 therapy with enhanced cytotoxic T-cell activity. Reports of the use of anti-IL2R to reverse PRCA and aplastic anemia were reviewed, but it was decided to observe initially, without reversing IL-2 effect, to evaluate antitumor response.

She continued to require red cell transfusions for 5 months following IL-2 therapy. She then became transfusion independent, maintaining her Hgb at 9.3 g/dL for several months. She declined a follow-up bone marrow evaluation. Subsequently, her hemoglobin improved spontaneously, normalizing to a level of 13.8 g/dL with Hct of 41.3% in June 2013 and remaining in the normal range (Hgb 13.9, Hct 42.6%; July 11, 2014).

Follow-up scans revealed a mixed response to IL-2 therapy, with progression of one lung lesion, but regression of the other and with no evidence of residual disease in the liver. She was referred to thoracic surgery for resection of the solitary progressing lesion. This was resected and she continued to be followed.

## Discussion

Interleukin-2 therapy has been associated with mild hematologic cytopenias, usually very short-lived, and occurring during or immediately following the actual treatment administration. This has included thrombocytopenia, neutropenia, and even a decline in hemoglobin, all of which reversed on completion of treatment.^19-22^ In most cases, transfusions were not required, although there did appear to be a dose-response relationship with the amount of IL-2 administered and the decrease in blood counts. Some patients who had had prior chemotherapy seemed to have deeper nadir counts as well.^19-22^ We evaluated bone marrows in cases of significant thrombocytopenia and these were normal with normal morphology and numbers of megakaryocytes, suggesting peripheral sequestration or destruction, which did not extend beyond completion of IL-2 therapy (J. P. Dutcher, personal observation). Additional hematologic toxicity during IL-2 therapy included acquired functional abnormalities of neutrophils, leading to chemotactic defects and increased risk of infection,^23,24^ and abnormalities of platelet function.^25^ An unusual coagulopathy was also observed, rarely leading to complications and possibly related to changes in hepatic synthesis.^26,27^ However, all of these changes reversed on completion of and recovery from IL-2 treatment.

In this patient, there were no other indications of causes of acute anemia. There was no evidence of bleeding or hemolysis. Although she had an IgG antibody to parvovirus B19, this is seen in 30% to 60% of adults, and in more than 80% of adults over the age of 50, and indicates previous exposure.^28-31^ Her IgM antibody titer was low and this combination precludes an acute infection, since elevated IgM titers should be concurrent with the cytopenias.^28^ Her hepatitis serologies were negative (hepatitis A, B, C and cytomegalovirus).

Recently, a report provided long-term follow-up of patients with moderate aplastic anemia and PRCA treated with daclizumab, a humanized monoclonal antibody to the IL-2 receptor.^31^ Daclizumab inhibits T-cell activation and is utilized in solid organ transplantation. In that report, among 27 evaluable patients with PRCA treated with this antibody, 6 achieved normalization of hemoglobin and another 4 had partial responses. Most required a second course of treatment, but with a median follow-up of 5.1 years, only 1 of the 6 complete responder patients had had a relapse and again responded to daclizumab. All were transfusion independent at the time of the report.^31^

Thus, alterations in cellular immunity are implicated in PRCA, and modulation of T-cells can affect this disease. A case of immune-mediated PRCA, among multiple immune adverse events, following treatment with a check-point inhibitor (anti-CTLA-4) has also been reported.^32^ In our patient, we utilized IL-2 to activate an anticancer cellular immune response and obtained a broad activation of immunity, leading to PRCA (she meets all diagnostic criteria). Fortunately, we did not need to implement immunosuppression, and we maintained the patient with supportive care. The PRCA resolved spontaneously over several months. She had a mixed response to the single course of IL-2 therapy, with resection of residual disease.

It should be noted that there is a single case report of a patient with PRCA that responded to corticosteroids, and was found to concurrently have a stage I renal cell carcinoma.^33^ However, this patient had had a thymoma resected 5 years previously. The literature reports delayed PRCA years after resection of benign thymoma.^34^ In our patient there was no anemia observed at the time of her nephrectomy, nor at the diagnosis of metastatic disease, but only following immune activation by high-dose IL-2. To our knowledge, this is the first case report of PRCA following high-dose IL-2 therapy.
